# Salivary creatinine as a diagnostic tool for evaluating patients with chronic kidney disease

**DOI:** 10.1186/s12882-019-1546-0

**Published:** 2019-10-29

**Authors:** Dada Oluwaseyi Temilola, Karla Bezuidenhout, Rajiv Timothy Erasmus, Lawrence Stephen, Mogamat Razeen Davids, Haly Holmes

**Affiliations:** 1 Division of Oral Medicine and Periodontics, Faculty of Dentistry, University of the Western Cape, Cape Town, South Africa; 2 Division of Nephrology, Department of Medicine, Stellenbosch University and Tygerberg Hospital, Cape Town, South Africa; 3 Division of Chemical Pathology, National Health Laboratory Service, Stellenbosch University and Tygerberg Hospital, Cape Town, South Africa

**Keywords:** Salivary creatinine, Serum creatinine, Chronic kidney disease

## Abstract

**Background:**

Preliminary studies have shown the potential use of salivary creatinine concentration in the diagnosis of chronic kidney disease (CKD). For saliva to replace serum as a diagnostic tool, studies must be done to determine its effectiveness in the diagnosis and staging of CKD. The aim of the present study was to evaluate the use of salivary creatinine as a safe and non-invasive alternative for identifying patients with CKD.

**Methods:**

A cross-sectional study was conducted at Tygerberg Hospital in Cape Town, on 230 patients, across all stages of CKD. Ethical approval to conduct the study was obtained from the University of the Western Cape Biomedical Research Ethics Committee, and written informed consent was provided by each participant. Saliva and serum samples were collected for creatinine analysis and the correlation determined using Spearman’s correlation. Receiver operating characteristics (ROC) analysis was used to determine the diagnostic ability of salivary creatinine. A cut-off value for optimal sensitivity and specificity of salivary creatinine to diagnose CKD with glomerular filtration rate (GFR) < 60 mL/min/1.73 m^2^ was obtained.

**Results:**

Serum creatinine values ranged from 46 μmol/L to 1581 μmol/L, with a median value of 134 μmol/L. Salivary creatinine values ranged from 3 μmol/L to 400 μmol/L, with a median of 11 μmol/L. There was a strong positive correlation (r = 0.82) between serum and salivary creatinine values. Linear regression analysis of serum and salivary creatinine for CKD patients was significant in all CKD stages, except for stage 1. Area under the curve for salivary creatinine was 0.839. A cut-off value of 8.5 μmol/L yielded a sensitivity of 78.3% and specificity of 74.0% for classifying patients as having CKD based on estimated GFR < 60 mL/min/1.73 m^2^.

**Conclusions:**

The results support the potential of salivary creatinine as a non-invasive diagnostic tool for estimating GFR and identifying patients with CKD.

## Background

Chronic kidney disease (CKD) is an important public health problem with an estimated global prevalence of 11 to 13% [1, 2]. The major drivers of this epidemic are the increases in new cases of diabetes mellitus and hypertension. CKD may lead to kidney failure and is also a strong risk factor for heart disease and stroke. In addition, there is a large impact on quality of life [3].

A systematic review of the burden of CKD in the general population and high-risk groups in Africa reported prevalences ranging from 2 to 41% [4]. A review of the burden of CKD in sub-Saharan Africa by Stanifer et al. estimated the population prevalence of CKD at 13.9% [5]. In South Africa, two population prevalence studies have been published. Matsha et al. reported a crude prevalence of 17.3% in a geographical cohort [6], while Adeniyi et al. reported an age-adjusted prevalence of 6.4% in a cohort of teachers [7].

The assessment of glomerular filtration rate (GFR) is an important part of the diagnosis and staging of CKD. Markers used to measure GFR include inulin, creatinine, urea and cystatin C. Creatinine is the most commonly used marker in clinical practice [8]. GFR is estimated from measurements of creatinine concentrations in blood, using various prediction equations. In adults, the Chronic Kidney Disease Epidemiology Collaboration (CKD-EPI) and the Modification of Diet in Renal Disease (MDRD) study equations are the most widely used [3]. A simple diagnostic test that does not require a blood sample and provides a reliable evaluation of CKD status would be of benefit to both patients and healthcare providers.

Saliva contains various components that may be used as biomarkers to detect systemic diseases or exposure to harmful substances. Salivary research is growing rapidly [9], due to the application of new scientific approaches such as bioinformatics, metabolomics, genomics and proteomics. Saliva has been shown to be useful in detecting various local diseases such as oral, head and neck cancers [10], lung, pancreatic, breast and ovarian cancers [11–14] and in the diagnosis of systemic diseases such as type 2 diabetes.

Recent studies have investigated the role of saliva as a substitute for serum or plasma in the diagnosis of CKD. These studies have mainly focused on its use in end-stage renal disease (ESRD) [15–17]. For saliva to replace blood as a diagnostic and monitoring tool for CKD, more studies must be conducted to demonstrate its effectiveness in diagnosing CKD and to classify patients into the correct CKD stage. Our study therefore investigated the correlation between serum and salivary creatinine levels to evaluate the role of saliva as a safe and non-invasive alternative for GFR estimation to identify patients with CKD.

## Methods

This cross-sectional study was conducted between February and May 2017 at the outpatient clinics of the Division of Nephrology at Tygerberg Hospital, a large public sector facility in Cape Town, South Africa. Patients 18 years and older who were willing to provide informed consent, were considered for inclusion in the study. Patients were excluded if they had any oral condition causing bleeding into the oral cavity.

### CKD diagnosis and staging

A GFR < 60 mL/min/1.73 m^2^ is used as an important cut-off for the diagnosis of CKD. This level defines stage 3 CKD. Stage 4 CKD is defined by GFR < 30 mL/min/1.73 m^2^ and stage 5 by GFR < 15 mL/min/1.73 m^2^. Stages 1 (GFR ≥ 90 mL/min/1.73 m^2^) and 2 (GFR 60–89 mL/min/1.73 m^2^) are diagnosed only when there are other markers of kidney damage present (e.g. proteinuria, imaging abnormalities, functional or histological abnormalities) [3].

### Sample size

The sample size was calculated using the estimated means of salivary creatinine in known test and control groups [18], with the level of significance set at 0.05 and the power at 90%. The calculated minimum sample size for each stage of CKD was rounded up to 40 participants, which provided for an attrition rate of 20%. CKD staging was based on participants’ estimated GFR (eGFR). Those with evidence of kidney disease for more than 3 months with GFR greater or equal to 90 mL/min/1.73 m^2^ were classified into stage 1; GFR between 60 and 89 mL/min/1.73 m^2^ were classified into stage 2; GFR between 30 and 59 mL/min/1.73 m^2^ was classified into stage 3; GFR between 15 and 29 mL/min/1.73 m^2^ was classified into stage 4 and those with GFR less than 15 mL/min/1.73 m^2^ were classified into stage 5. Patients on dialysis were excluded from the study.

### Saliva and blood sampling

Saliva collection was carried out between 09:00 am and 12:30 to minimize the effect of diurnal variation. Participants were instructed to refrain from eating and drinking at least 90 min before collection and to thoroughly rinse their mouths with water, prior to the sample collection, to void the mouth of saliva. Two mL of whole saliva was collected in a sterile graduated container by the spitting method. Participants were asked to sit in a comfortable position with eyes open and head tilted slightly forward and to avoid swallowing or other oral movements during collection. The pooled saliva in the floor of the mouth was spat into the container every 60 s or just before they experienced an urge to swallow the accumulated fluid. This process was repeated until 2 mL of whole saliva was obtained.

Immediately after saliva sample collection, 2 mL of blood was collected from patients’ antecubital veins in serum separator tubes. Blood samples were allowed to clot at room temperature for one hour and then centrifuged at 1000 g for 10 min at 4 °C. The saliva samples were centrifuged at 1000 g at for 10 min and the supernatant obtained was stored at − 80 °C prior to final analysis. Creatinine levels in saliva and serum were analyzed on a Roche cobas® 6000 analyzer, which uses an enzymatic method for creatinine analysis.

### Statistical analysis

Data were entered into MS Excel and analyzed using SPSS v17.0. Spearman’s correlation coefficient (r) was used to test the correlation between serum and salivary creatinine levels. Linear regression equations were derived to estimate the level of serum creatinine from the salivary levels. Receiver operating characteristic (ROC) analysis was used to evaluate the diagnostic potential of salivary creatinine compared to serum creatinine. The overall performance was assessed by the total area under the curve and the cut-off values were determined based on the best trade-off between the sensitivity and specificity.

### Ethics approval and consent to participate

Approval to conduct the study was obtained from the Biomedical Research Ethics Committee of the University of the Western Cape (project number BM/16/5/4). All participants provided written informed consent.

## Results

The number of participants included in stages 1, 2 and 3 was increased to 50 each as these stages were the most prevalent. Stages 4 and 5 included 40 participants each and the final study cohort therefore comprised a total of 230 patients. There were more female participants overall (61.3%) and within each CKD group (Table 1).

**Table 1:** The frequency distribution of patients by CKD stages, gender, age and comorbidities

Gender	Stage 1	Stage 2	Stage 3	Stage 4	Stage 5	Total
Male	16	17	23	19	14	89 (38.7%)
Female	34	33	27	21	26	141 (61.3%)
All	50	50	50	40	40	230 (100%)
Age range Median age Interquartile range (Q1-Q3)	18–54 31.0 (21.25-39.75)	19–73 38.5 (31.25-49.50)	19–73 38.0 (31.0-51.0)	20–82 40.5 (33.75-47.5)	21–66 47.5 (40.5-54.25)	
Patients with co-morbidities	34	47	44	39	40	204
	Hypertension and diabetes (2)	Hypertension and diabetes (7)	Hypertension and diabetes (5)	Hypertension and diabetes (4)	Hypertension and diabetes (10)	
	Hypertension (28)	Hypertension (36)	Hypertension (34)	Hypertension (32)	Hypertension (29)	
	Diabetes (1)	Diabetes (2)	Diabetes (2)	Diabetes (1)	Diabetes (0)	
	Other (3)	Other (2)	Other (3)	Other (2)	Other (1)	

**Fig. 1:**
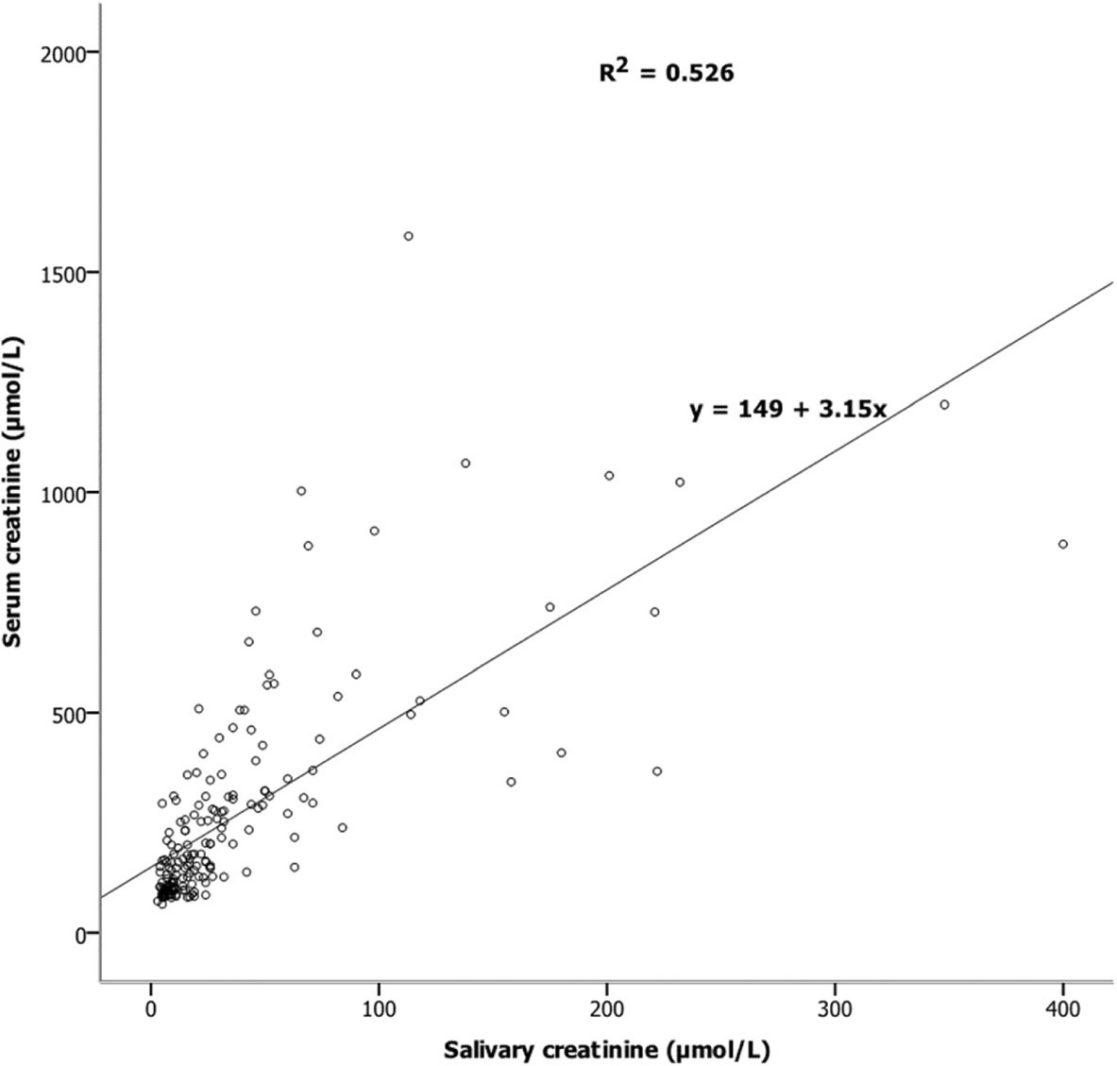
Linear regression between serum and salivary creatinine levels of all CKD patients

The ranges of serum and salivary creatinine values were 46–1581 μmol/L and 3–400 μmol/L, respectively. Table 2 shows the ranges and medians for CKD stages 1 to 5, and for all patients combined. There was a strong positive correlation (r = 0.82) between serum and salivary creatinine when considering all samples, and a moderate correlation for patients in CKD stages 2 to 5 (Table 3). Linear regression analysis demonstrated an ability to predict the serum creatinine from salivary creatinine in CKD stages 2 to 5 (Fig. 1 and Table 4).

**Table 2:** Serum and salivary creatinine (in μmol/L) for patients in CKD stages 1–5

	Stage 1	Stage 2	Stage 3	Stage 4	Stage 5	All
Serum creatinine: (median) range	46–93 (66)	65–133 (91)	102–232 (149)	161–462 (276)	312–1581 (518)	46–1581 (134)
Salivary creatinine: (median) range	3–19 (6)	3–18 (9)	4–63 (16)	5–222 (29)	72–40 (68)	3–400 (11)

**Table 3:** Spearman correlation analysis of serum and salivary creatinine

	All patients	Stage 1	Stage 2	Stage 3	Stage 4	Stage 5
*R*	0.82	0.16	0.31	0.38	0.42	0.55
*P*	< 0.001	0.250	0.030	0.005	0.007	< 0.001

Correlation is considered strong at (*r* = 1.0 to 0.5), moderate at (*r* = 0.3 to 0.5), weak at (*r* = 0.1 to 0.3), very weak or no correlation at (*r* < 0.1)

**Table 4:** Linear regression analysis to predict serum creatinine from salivary creatinine in CKD patients stages 1 to 5

Stage	*p*-value	Linear regression equation	*R* ^*2*^	*R*^*2*^ (%)	R
Stage 1	0.465	Y = 64.9 + (0.23) × (salivary Cr)	0.008	0.80%	0.894
Stage 2	0.030	Y = 89.7 + (0.54) × (salivary Cr)	0.035	3.50%	0.187
Stage 3	0.008	Y = 134 + (0.95) × (salivary Cr)	0.140	14.0%	0.374
Stage 4	0.007	Y = 251 + (0.6) × (salivary Cr)	0.179	17.9%	0.423
Stage 5	0.001	Y = 451 + (1.72) × (salivary Cr)	0.275	27.5%	0.524

**Fig. 2:**
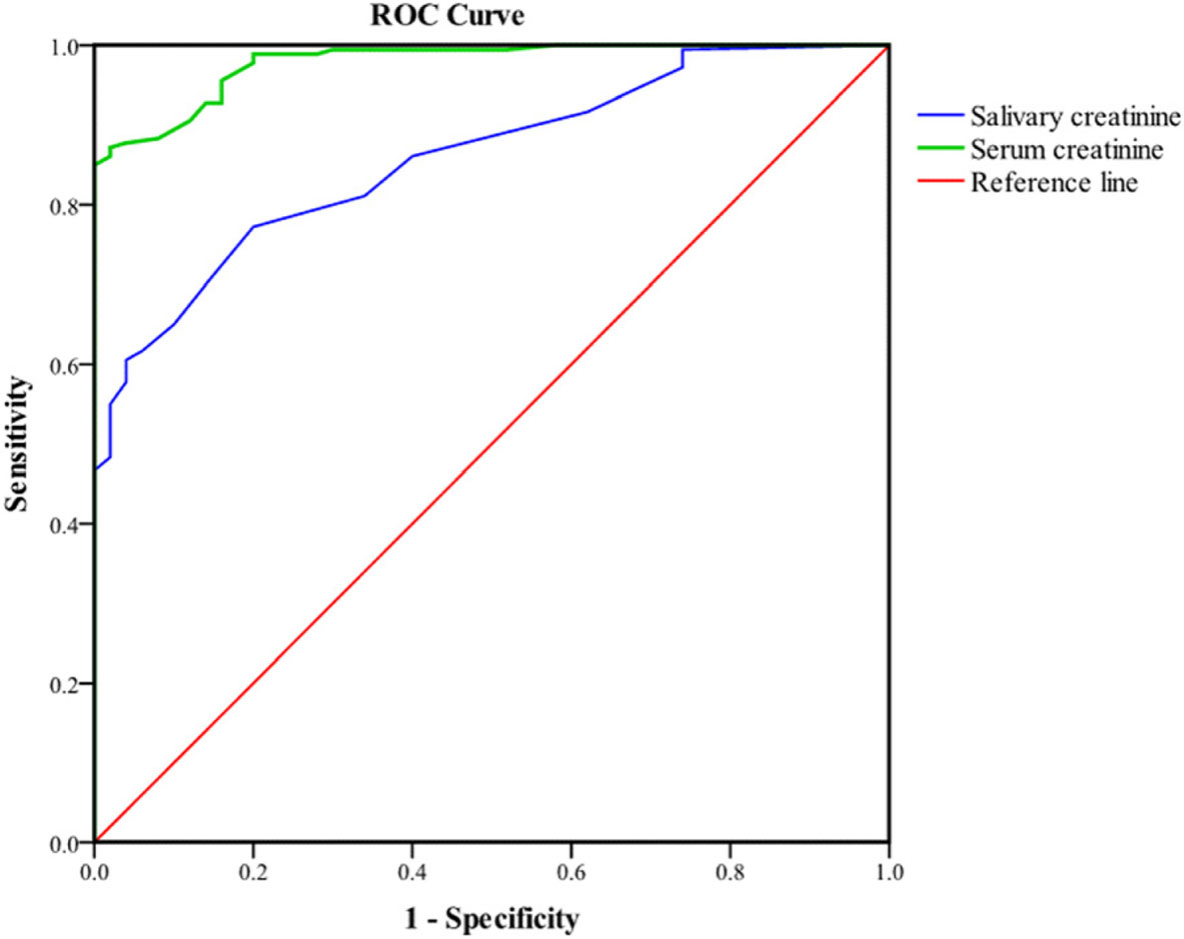
ROC curve of serum and salivary creatinine levels

ROC analysis (Fig. 2) found the total area under the curve to be 0.89 (standard error = 0.028, *p*-value < 0.001, 95% confidence interval = 0.784–0.894). Table 5 shows the sensitivity and specificity for different values of salivary creatinine; 8.50 μmol/L was determined as the best cut-off value to diagnose CKD based on a GFR value < 60 mL/min/1.73 m^2^. This cut-off point yielded a sensitivity of 78.3% (false negative rate 21.7%), a specificity of 74.0% (false positive rate 26%) and a positive predictive value (PPV) of 79.6%.

**Table 5:** Sensitivity and specificity analysis of salivary creatinine for different cut-off values to diagnose CKD on the basis of GFR < 60 mL/min/1.73 m^2^

Salivary creatinine (μmol/L)	Sensitivity (%)	Specificity (%)
3.5	99.4	26.0
4.5	97.2	26.0
6.5	86.1	56.0
8.5	78.3	74.0
11.5	64.4	88.0
14.5	60.6	90.0
15.5	57.8	92.0

## Discussion

The present study reported a progressive increase in salivary creatinine levels from CKD stages 1–5, consistent with the findings of other studies [15–17], and a strong overall correlation between serum and salivary creatinine. Lloyd et al. reported a similar pattern in patients with CKD stages 4 and 5 [19]. Linear regression also demonstrated a significant relationship between serum and salivary creatinine in CKD stages 2 to 5, with stage 5 having the highest coefficient of determination. This is in line with previous studies in which a significant predictive relationship was found between serum and salivary creatinine in CKD stages 4 and 5. For CKD stage 1, the relationship between serum and salivary creatinine was not significant; this is also consistent with results of previous studies [16, 17] and is thought to be due to the lower serum creatinine levels, with minimal movement of creatinine to saliva due to the lack of a large concentration gradient.

Salivary creatinine can only be accepted as an alternative diagnostic method if it is comparable to serum creatinine in its ability to differentiate between those with and without CKD [20]. ROC analysis showed a large area under the curve for salivary creatinine, suggesting that it may be a good alternative diagnostic test to identify CKD patients with GFR below the critical value of 60 mL/min/1.73 m^2^. Comparable large areas under the curve were reported in previous studies. Xia et al. [21] obtained an area under curve value of 0.897, while Ventakapathy et al. [17] obtained a value of 0.967.

In the present study, the optimal cut-off point for the diagnosis of CKD was determined to be a salivary creatinine concentration of 8.5 μmol/L, which provided good sensitivity and specificity, and a high positive predictive value (PPV) in our cohort of participants. This suggests that salivary creatinine could be used as a non-invasive tool in diagnosing CKD and that people with values above 8.5 μmol/L should be referred for further diagnostic evaluation and appropriate management. A study by Renda et al. [22] reported a cut-off value of 0.125 mg/dl (11.1 μmol/L) as the optimum salivary concentration for diagnosis of CKD among children in stage 2–5. Ventakapathy et al. [17] studied patients with stage 4 and 5, and reported a cut-off value of 0.2 mg/dl (17.7 μmol/L). The higher cut-off value reported by Ventakapathy et al. may be due to the consideration of only CKD stages 4 and 5, compared to the present study which considered patients in stages 1–5. Correlation studies of serum and salivary creatinine in healthy individuals have reported conflicting results. Bader et al. [15] found a positive correlation between serum and salivary creatinine in patients without kidney disease, while other studies have reported negative correlations [16, 17]. These differences may be due to the presence of factors such as diabetes mellitus, hypertension and salivary gland diseases, which can influence the diffusion of creatinine from serum into the salivary glands [23, 24]. Another potential confounder is medication use, which may alter salivary gland cell permeability and thereby salivary creatinine concentrations [25]. Other markers which have been used to estimate renal function include salivary urea, which was first used in the early part of the twentieth century [26]. Its concentration is more affected by non-renal factors than is the case with creatinine [27]. These factors include hydration status, protein intake, protein catabolism, liver diseases, gastrointestinal bleeding, and therapy with high-dose steroids [27]. In view of these limitations, and because serum creatinine is commonly used to estimate GFR, we focused on this marker in the present study. It may be that a combination of several salivary markers may improve the accuracy of assessing renal function and this needs to be tested in future studies.

Limitations of this study include the use of estimated GFR for evaluating renal function, rather than measured GFR as determined by the clearance of iohexol or other exogenous markers. Only a single salivary sample was taken from each patient and we had an absence of healthy controls. Considering that the sample was drawn from a nephrology clinic, the proportion of subjects with CKD is much higher than would be expected in a CKD screening study of the general population. Additional studies are needed to test the utility of salivary creatinine in these settings where most of the subjects would be expected to have normal or near-normal concentrations of serum creatinine. Future studies should investigate whether salivary creatinine is correlated with other clinical and laboratory parameters, and should examine the utility of newer markers of kidney damage, such as NGAL,

## Conclusion

The present study has contributed to the existing data supporting the diagnostic potential of salivary creatinine as a non-invasive tool to estimate GFR. Salivary creatinine concentrations above 8.5 μmol/L may identify patients with CKD and should prompt referral for further diagnostic evaluation.

## Data Availability

The datasets generated during the current study are available from the corresponding author on request.

## Abbreviations

CKD: Chronic kidney disease
eGFR: estimated glomerular filtration rate
NPV: Negative predictive value
PPV: Positive predictive value
ROC: Receiver operator characteristics
